# Comparative analysis of population with positive anti-gliadin antibody and anti-Saccharomyces cerevisiae antibody

**DOI:** 10.12669/pjms.42.3.12767

**Published:** 2026-03

**Authors:** Xin Liu, Mingyue Zhang, Yue Hu, Kai Yang, Sha Feng, Wei Geng

**Affiliations:** 1 Xin Liu Department of Clinical Laboratory, Baoding No.1 Central Hospital, Baoding 071000, Hebei, China; 2 Mingyue Zhang Department of Molecular Biology Laboratory, Baoding People’s Hospital, Baoding 071000, China; 3 Yue Hu Department of Clinical Laboratory, Baoding No.1 Central Hospital, Baoding 071000, Hebei, China; 4 Kai Yang Department of Emergency, Baoding No.1 Central Hospital, Baoding 071000, Hebei, China; 5 Sha Feng Department of Clinical Laboratory, Baoding No.1 Central Hospital, Baoding 071000, Hebei, China; 6 Wei Geng Department of Cardiovascular Medicine, Baoding No.1 Central Hospital, Baoding 071000, Hebei, China

**Keywords:** Anti-gliadin antibody, Anti-Saccharomyces cerevisiae antibody, Distribution characteristics, Rate of misdiagnosis and missed diagnosis

## Abstract

**Objective:**

To explore the distribution characteristics of population with positive anti-gliadin antibody (AGA) and anti-Saccharomyces cerevisiae antibody (ASCA), reveal the important value of early detection of AGA and ASCA.

**Methodology:**

This is a retrospective comparative analysis. Comparative analysis of AGA and ASCA detected data in Baoding No.1 Central Hospital from February 2021 to February 2024. The rate between groups was compared by χ^2^ test. *P*<0.01 was considered as statistically significant.

**Results:**

The ratio of male to female was 1:1.4. The number of male patients with AGA positive was significantly lower than that of females (χ^2^=88.76, *P*<0.01). Among the 1286 patients with ASCA positive, there were 726 males and 560 females. The ratio of male to female was 1:0.77. The number of male patients with AGA positive was significantly higher than that of females (χ^2^=42.86,*P*<0. 01). There are great differences in the distribution of AGA and ASCA between different genders. With the increase of age, the positive rate of AGA showed a downward trend and ASCA showed an upward trend. There are great differences in the distribution trend of AGA and ASCA with age.

**Conclusion:**

Early detection of AGA and ASCA have definite guiding significance for early diagnosis and differential diagnosis of digestive diseases to reduce misdiagnosis and missed diagnosis.

## INTRODUCTION

Gliadin is the skeleton protein of plant fruit wheat.^1^ Anti-gliadin antibody (AGA) is the product of the body’s allergic reaction and is an immunoglobulin secreted by B lymphocytes from the immune system that reacts against gliadin.^2^ The study have found that keratin and reticulin, which are the components of human cytoskeleton, are structurally similar to wheat collagen and have the same antigenic components.^3^ Once AGA is produced, it can cross-react with keratin and reticulin, damage its structure and cause intestinal wall tissue hardening, villus atrophy and so on. AGA can affect the function of keratin and reticulin, resulting in nausea, vomiting, anorexia, diarrhea and other digestive and malabsorption diseases. It is reported that the disease caused by AGA is gluten sensitive enteropathy, and it is also called celiac disease. In the past, it was considered that the incidence of celiac disease was low in China, but with the popularization and application of autoantibody detection technology in China, the diagnosis rate of celiac disease is increasing rapidly.^4^

Saccharomyces cerevisiae, also known as baker’s yeast,^5^ is a commonly used biological species in fermentation, which widely exists in nature. The target antigen of anti-Saccharomyces cerevisiae antibody (ASCA) is phosphopeptide mannan,^6^ which is the main component of cell wall of Saccharomyces cerevisiae. ASCA is also a product of allergic reactions. Once ASCA is produced, it can damage the intestinal mucosa, causing abdominal pain, diarrhea, granuloma and even polyps. The disease caused by ASCA is called Crohn’s disease. The common clinical symptom of Crohn’s disease is long-term abdominal pain with diarrhea. Due to the lack of specificity, it is easy to misdiagnose and delay the disease.^7^ Based on this, this study explored the distribution characteristics of anti-gliadin antibody (AGA) and anti-brewing yeast antibody(ASCA) positive populations, revealing the clinical value of early detection of AGA and ASCA.

## METHODOLOGY

This is a retrospective comparative analysis. AGA and ASCA were detected in 6969 patients in the department of gastroenterology, Baoding NO.1 Central Hospital from February 2021 to February 2024, including 3435 males and 3534 females, with an age range of 1-96 years old.

### Ethics approval:

The study was approved by the Institutional Ethics Committee of Baoding No.1 Central Hospital (No.: [2021]092; date: October 13, 2021), and written informed consent was obtained from all participants.

### Inclusion criteria:

- Patients who seek medical treatment in the gastroenterology department of our hospital;
- Informed consent to the study and having complete clinical data.

### Exclusion criteria:

- Suffering from severe organ dysfunction such as heart, liver, kidney, and other immune related diseases;
- Accompanied by coagulation dysfunction;
- With malignant tumors or psychological and psychiatric disorders.

All data of these patients were collected from the electronic medical record system of our hospital. Retrospective analysis and comparative analysis of AGA and ASCA detected data in our hospital in the past three years. The levels of AGA and ASCA in venous serum were detected by immunoblotting. The testing of AGA and ASCA strictly follows the standard operating procedures.

### Statistical analysis:

Statistical analysis was performed using SPSS Version 20.0 software (IBM Corp., Armonk, NY, USA). The counting data of between groups presented in the form of (n, %) and compared by χ^2^ test. The difference was statistically significant with P< 0.05.

## RESULTS

In the past three years, A total of 6969 cases of AGA and ASCA were simultaneously detected in our hospital, of which 1535 cases were positive for AGA with a positive rate of 22.03%; 1286 cases were positive for ASCA with a positive rate of 18.45%; 231 cases were positive for AGA and ASCA with a positive rate of 3.31%. The positive rate of patients with both AGA and ASCA positive was lower. Among the 1535 patients with AGA positive, 637 were male (41.50%), and 898 were female (58.50%). The ratio of males to females was 1:1.4. The number of females in AGA-positive patients was significantly higher than that of males. The difference was statistically significant(χ^2^=88.76, P<0.01). Among the 1286 patients with ASCA positive, 726 were male (56.45%), and 560 were female (43.55%). The ratio of males to females was 1:0.77. The number of males in ASCA-positive patients was significantly higher than that of females. The difference was statistically significant(χ^2^=42.86, P<0.01). There were significant differences in the distribution of AGA and ASCA among different genders (Table-I).

**Table-I:** Comparative analysis of AGA and ASCA positive rates with gender.

	Cases	Males	Females	χ^2^	P*
		Cases/Positive rate%	Cases/Positive rate%		
AGA Positive	1535	637(41.50)	898(58.50)	88.76	<0.001
ASCA Positive	1286	726(56.45)	560(43.55)	42.86	<0.001

***Note:***

* All applied Chi-square test.

**Fig.1:**
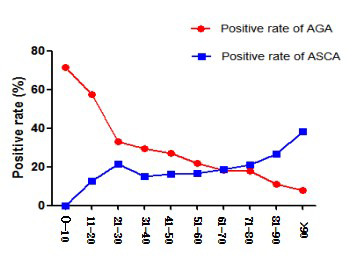
Comparative analysis of the distribution trend of AGA and ASCA positive rate with age.

The participants were divided into one group every 10 years old, and the positive rates of AGA and ASCA were compared and analyzed. We found that with the increase of age, the positive rate of AGA showed a downward trend, and the positive rate of ASCA showed an upward trend. In ≤60 years old group, the positive rate of AGA was higher than that of ASCA. In >60 years old, and the positive rate of AGA was lower than that of ASCA. There were significant differences in the distribution of AGA and ASCA with age (Table-II, Fig.1).

**Table-II:** Comparative analysis of AGA and ASCA positive rates with age.

Age	Cases	AGA Cases/Positive rate%	ASCA Cases/Positive rate%	χ^2^	P*
0-10	21	15(71.43)	0(0)	23.33	<0.001
11-20	120	69(57.50)	15(12.50)	53.41	<0.001
21-30	228	75(32.89)	49(21.49)	7.49	0.006
31-40	555	163(29.37)	83(14.95)	33.42	<0.001
41-50	1026	277(27.00)	166(16.18)	35.47	<0.001
51-60	1601	349(21.80)	270(16.86)	12.50	<0.001
61-70	1854	341(18.39)	345(18.61)	0.03	0.866
71-80	1111	197(17.73)	233(20.97)	3.74	0.053
81-90	414	46(11.11)	110(26.57)	32.35	<0.001
>90	39	3(7.69)	15(38.46)	10.40	0.001
Total	6969	1535(22.03)	1286(18.45)	27.56	<0.001

***Note:***

* All applied Chi-square test.

### Upper gastrointestinal symptoms:

Patients repeatedly appeared one or more upper gastrointestinal symptoms such as epigastric discomfort, nausea and vomiting, heartburn, regurgitation of gastric acid, loss of appetite and other upper gastrointestinal symptoms. Lower gastrointestinal symptoms: Patient repeatedly appeared one or more lower gastrointestinal symptoms such as abdominal pain, diarrhea, abnormal defecatel, falling lower abdomen and other lower gastrointestinal symptoms.

Among 1535 cases of AGA positive patients, 1099 cases (including 83 cases of patients with lower gastrointestinal symptoms) had upper gastrointestinal symptoms, accounting for 1099 / 1535 = 71.60%; 522 cases (including 83 cases of patients with upper gastrointestinal disease) had lower gastrointestinal symptoms, accounting for 522 / 1535 = 34.01%; 83 cases had both upper and lower gastrointestinal symptoms, accounting for 83/1535=5.38%.

Among 1286 cases of ASCA positive patients, 431 cases (including 51 cases of patients with lower gastrointestinal symptoms) had upper gastrointestinal symptoms, accounting for 431/1286 =33.51%; 868 cases (including 51 cases of patients with upper gastrointestinal disease) had lower gastrointestinal symptoms, accounting for 868/1286=67.50%; 51 cases had both upper and lower gastrointestinal symptoms, accounting for 51/1286=3.97%.

The incidence of upper gastrointestinal symptoms in AGA positive group was higher than that in ASCA positive group, and there was significant difference between the two groups(χ^2^= 408.85, P < 0.01). The incidence of lower gastrointestinal symptoms in AGA positive population was lower than that in ASCA positive population, and the difference was statistically significant(χ^2^= 406.92, P < 0.01) (Table-III).

**Table-III:** Comparative analysis of clinical characteristics of AGA and ASCA positive population.

	AGA positive	ASCA positive	χ^2^	P*
Upper gastrointestinal symptoms	1099(71.60)	431(33.51)	408.85	<0.001
Lower gastrointestinal symptoms	522(34.01)	868(67.50)	313.99	<0.001

***Note:***

* All applied Chi-square test

## DISCUSSION

Based on the investigation and analysis of large sample AGA test data of patients in the department of gastroenterology, we found that the positive rate of AGA was as high as 22.03%, which was very close to the positive rate of 20.7% reported by Cao Z et al.^8^,^9^ AGA is the product of allergic reaction, and its formation is greatly affected by environmental factors.^10^ The great changes of diet structure in China are closely related to the generation of AGA. The disease caused by AGA is gluten sensitive enteropathy. We have a late understanding of this kind of disease, especially lack of understanding of the pathogenicity of AGA. In particular, the lack of popularization of fundamental laboratories testing has led to a high rate of misdiagnosis and missed diagnosis of these diseases, which seriously affects the health of population. Fully understanding the mechanism of AGA and the necessity of laboratory detection are of great significance for the early prevention and treatment of diseases induced by AGA. This survey found that the positive rate of ASCA in patients in the department of gastroenterology was 18.45%, which was very close to the positive rate of ASCA in patients with intestinal infection, intestinal tuberculosis, intestinal polyps and other diseases reported by Vermeire et al.^11^ It is much higher than the report that the positive rate of ASCA in healthy people is 1-7%.^5^ The sensitivity of ASCA to Crohn’s disease reached 65.5%.^12^ Crohn’s disease and ulcerative colitis are difficult to distinguish, generally referred to as “inflammatory bowel disease”. However, with the improvement of autoantibody detection technology in recent years, the clinical differentiation of the two kinds of diseases has been simple and clear. The occurrence of ulcerative colitis is related to the action of antineutrophil cytoplasmic antibody (ANCA), an important member of autoantibodies, on the intestinal wall.

In this survey, the proportion of patients with both AGA and ASCA positive was only 3.31%, We did not find that there was a significant co-effect between AGA and ASCA. The positive rates of AGA and ASCA positive populations vary greatly with gender and age distribution. The positive rate of AGA in female was higher than that in male, and the ratio of male to female was 1:1.4. The positive rate of AGA decreased with age. Epidemiological survey found that the incidence of gluten-sensitive enteropathy in children and adolescents was high, the ratio of male to female is about 1:1.3-2.0.^13^ It has been reported that the sensitivity of AGA decreased with age,^14^ which is consistent with the positive distribution of AGA in our study. As a serum marker of gluten-sensitive enteropathy, the positive distribution of AGA is consistent with the incidence of gluten-sensitive enteropathy. The high incidence of gluten -sensitive enteropathy in adolescents should be related to a variety of congenital and acquired factors.^15^ Previous studies have shown that AGA can be found in the serum of asymptomatic individuals in adolescents.^16^ AGA has the value of disease early warning. Early detection of AGA and adherence to a gluten-free diet for patients with AGA positive have a significant effect on the early prevention and treatment of disease.

Ye et al.^17^ reported that the ratio of men to women in Crohn’s disease is about 1.5:1 in China, which was consistent with our survey results on the distribution of ASCA-positive people with gender. Israeli et al.^18^ also found that 31% of patients with Crohn’s disease had ASCA in their serum before they were diagnosed, and the positive rate of ASCA gradually increased with the progress of Crohn’s disease. ASCA has predictive value for diseases, and early detection is of great significance. AGA-related diseases are caused by allergic reactions to antigenic components in food. Reticulin, keratin and other components that cross-react with AGA are also widely found in gastric parietal cells tissue. Therefore, in addition to intestinal mucosal injury, AGA can also cross-react with gastric tissue components to damage its structure and function, resulting in upper gastrointestinal related symptoms. ASCA can cross-react with gut microbiota^19^ and cause chronic inflammatory reaction in intestinal tract. The gut microbiota in adults is mainly distributed in the colon and the end of the small intestine.^20^ Crohn’s disease mainly occurs in the colon and ileum. This survey showed that abdominal pain, diarrhea, intestinal polyps and other lower gastrointestinal symptoms were common in ASCA positive people, accounting for 67.50%.

The pathological manifestations of diseases caused by antibodies such as AGA, ASCA and ANCA are mostly lymphocyte infiltration, which is difficult to distinguish from intestinal lymphoma, intestinal tuberculosis and even malignant tumors in the early stage.^21^ It is necessary to detect antibodies such as AGA, ASCA and ANCA in time for patients in department of gastroenterology. This kind of antibody is traumatic and appears earlier than the typical symptoms in the patient’s serum.^21^ Timely detection not only has the value of disease early warning, but also can quickly and accurately identify a variety of digestive system diseases, so as to take effective measures to control the progress of the disease and reduce the rate of misdiagnosis and missed diagnosis.

The rate of misdiagnosis and missed diagnosis of inflammatory bowel disease remains high. Qian Jiaming, director of Peking Union Medical College Hospital, pointed out in his article that Meta-analysis showed that the misdiagnosis rate and missed diagnosis rate of Crohn’s disease are as high as 36.8% and 60.9%, respectively. The misdiagnosis rate and missed diagnosis rate of ulcerative colitis are 27.5% and 32.1% respectively.^22^ It can be seen that timely discovery and accurate diagnosis have a long way to go.

### Limitations:

Nevertheless, shortcomings can still be seen in this study: no long-term follow-up was performed and a limited sample size was included. In this regard, corresponding measures should be taken in the future to improve this study.

## CONCLUSIONS

The positive rates of AGA and ASCA in patients in the department of gastroenterology were higher, but their distribution characteristics with age and genders were quite different.

### Authors’ Contributions:

**XL** and **MZ:** Designed this study and prepared this manuscript.

**YH** and **KY:** Collected and analyzed clinical data.

**SF** and **WG:** Participated in acquisition, analysis, or interpretation of data and draft the manuscript.

All authors have read, approved the final manuscript and are responsible for the integrity of the study.
